# The influencing factors of elder-friendly public open spaces promoting older adults’ health in deprived urban neighborhoods: Partial Least Square Structural Equation Modeling approach

**DOI:** 10.3389/fpubh.2023.1143289

**Published:** 2023-04-17

**Authors:** Azadeh Lak, Soheila Khodakarim, Phyo K. Myint, Hamid R. Baradaran

**Affiliations:** ^1^Faculty of Architecture and Urban Planning, Shahid Beheshti University, Tehran, Iran; ^2^Department of Biostatistics, School of Medicine, Shiraz University of Medical Sciences, Shiraz, Iran; ^3^Ageing Clinical and Experimental Research Team, Institute of Applied Health Sciences, University of Aberdeen, Aberdeen, United Kingdom; ^4^Department of Epidemiology, School of Public Health, Iran University of Medical Sciences, Tehran, Iran

**Keywords:** public open space (POS), elderly health, perceived attributes, PLS-SEM, neighborhoods

## Abstract

**Background:**

Public open spaces (POSs) is considered a feature of the built environment that is important for physical, mental, and social health during life and contributes to active aging. Hence, policymakers, practitioners, and academics have recently focused on indicators of elder-friendly environments, particularly in developing countries.

**Objective:**

This study aimed to examine the attributes of POSs and socio-demographic status that positively influence older people’s health in Tehran’s deprived neighborhoods using a pathway model.

**Methods:**

We employed a pathway model to explore the relationships between place function, place preferences, and process in the environment as the perceived (subjective) positive features of POSs associated with older adults’ health, compared to the objective attributes of POSs. We also included personal characteristics, including physical, mental, and social dimensions, to explore how these factors are related to the health of older adults. To assess the subjective perception of POSs attributes, 420 older adults were asked to complete Elder-Friendly Urban Spaces Questionnaire (EFUSQ) from April 2018 to September 2018 in the 10th District of Tehran. We used the SF-12 questionnaire and “The self-Rated Social Health of Iranians Questionnaire to measure older people’s physical and mental health and elder social health.” Geographical Information System (GIS) measures (Street connectivity, Residential density, Land use mix, Housing quality) were derived as objective measures of neighborhood features.

**Results:**

According to our findings, the personal aspect, socio-demographic status (such as Gender, Marital status, Education, Occupation as well as Frequency of being present in POSs), place preferences (Security, Fear of Falling, Way Finding and Perceived Aesthetics), and process in the environment’s latent (Social Environment, Cultural Environment, Place Attachment, and Life Satisfaction)constructs collectively influenced the elders’ health.

**Conclusion:**

We found positive associations between Place preference, Process-in-environment, and personal health-related factors to elders’ health (social, mental, and physical). The path model presented in the study could be guided in future research in this area and inform the development of evidence-based urban planning and design interventions for improve older adults health and social functioning and quality of life.

## Introduction

The number of people above 65, i.e., older adults, will double by 2050 (1). Physiologically speaking, aging results from accumulated molecular and cellular damage, gradually decreasing physical and mental capacities and increasing chronic diseases (2). There is growing evidence to support late-life living environments as well as associations between the features of the outdoor environment and health outcomes (3–5). Well-maintained public open spaces (POSs) can enhance older adults’ physical and mental health and well-being (6–8). Changes in mortality, physical function, disease prevalence, and mental health status in different environments can support this relationship (9). With other attributes of the building, say, walkability, and public transport infrastructure, POSs bring about various benefits, such as promoting physical activity (7, 10) and creating social cohesion in neighborhoods ((10, 11); (7)). Older adults’ health depends on some elements, including lifestyle, genetic predisposition, social relationships, and environment (7).

Besides, the research found an association between POSs and chronic health conditions like obesity (4, 7), cardiovascular disease (12), diabetes (7, 12), respiratory health (13), and mental health (e.g., stress, anxiety, depression, and attention) deficit disorders (7, 12). In line with this body of research, POS in this study refers to local green spaces in neighborhoods.

Studies of older adults have found that some characteristics of POSs are likely to increase walking activities in residential neighborhoods (14–16). These characteristics include walking for transportation (17), recreational physical activity (15) in local streets (18), and urban parks (19, 20), including amenities like shade structures, toilets, trees and plants, and well-paved surfaces and like to experience minimal traffic and seat along walkways (1), “trees/plant” (20) and park seating (21).

The provision of appropriate POSs is a major social determinant of health contributing to an area’s livability, particularly in deprived urban neighborhoods (22), regarded as “safe, secure, attractive, socially cohesive and inclusive, and environmentally sustainable; with affordable and diverse housing linked to employment, education, public open space, local shops, health and community services, and leisure and cultural opportunities; *via* convenient public transport, walking and cycling infrastructure” (23).

To examine the impact of individual and environmental factors on the presence of older adults in POSs and on their health outcomes, our study adopts such an ecological perspective (24), which includes three potential mechanisms of older adults’ health as physical activity in outdoor spaces, social interaction, and contact with nature (25).

As Iranian society’s traditional structure disapproves of sending senior people to nursing homes, aging in place is the appropriate solution to help older adults in Iran remain physically and mentally healthy. Creating elder-friendly POSs can help them live active and healthy life and experience an acceptable quality of life (26). Although many researchers have already studied the relationship between POSs and older adults’ health, there is still a shortage of robust evidence to support contemporary policy demands in an aging society (12). Besides, the health status of older adults in the POSs of neighborhood’ was addressed internationally (7, 23), but there is a significant gap in Iran’s quantitative research (27, 28). A 2015 report by Tehran Municipality states that although Iran is a group of 33 countries involved in moving toward age-friendly cities, the government is not equipped with the necessary infrastructure (29–31). The collected data show that theoretical findings have led to neither a deep understanding of the local preferences of older adults in Iran nor practical guidelines for designing and developing age-friendly POSs. With this in mind, this study aims to explore the relationship between self-related health and features of POSs to enhance the presence of the elderly in POSs in the deprived neighborhoods of Tehran, Iran.

## Materials and methods

### Study site

This study was carried out in public spaces (POSs), including parks, squares, and neighborhood hangouts, in Tehran’s 10th district. Tehran’s 10th district has relatively the highest population among Tehran’s regions and the highest number of older adults. It has a relatively low area of residential use; therefore, most older adults use public spaces in their neighborhoods (32). Of 287,476 people in the 10th district of Tehran, more than 10 percent are above 60 years old (33).

### Study design

This cross-sectional study was conducted on 420 older people who regularly used public outdoor spaces (POSs) in Tehran’s 10th district neighborhoods over 4 months during Spring/Summer in 2018. Participants were asked to complete three questionnaires, the SF-12 questionnaire, the Self-Rated Social Health of Iranians Questionnaire, and an elder-friendly urban spaces questionnaire (EFUSQ), after obtaining their written consent assisted by research assistants. Trained research assistants facilitated this through face-to-face interviews. They all received a three-hour training which covered how to introduce questionnaires and administer interviews to ensure standardized facilitation for accuracy, completeness, and reliability. Data collection for each participant took about 20–30 min. The Iran University of Medical Sciences (IUMS) granted ethical approval, and all participants signed informed consent before participating in this research.

### Participants

From April through September 2018, participants were chosen using a purposeful (non-random) sampling technique. To be considered, the individuals had to frequent the open areas (parks, squares, and streets) in the District 10 neighborhoods of Tehran at least three times each week. Those with severe physical disabilities who could not use POSs and had communication problems were not considered for this study.

### Measurements

The subjective measure of the neighborhood’s perceived environment, which we used here, was measured through an adapted version of the Elder-Friendly Urban Spaces Questionnaire (EFUSQ) (28). The questionnaire consists of 50 items divided into three parts in terms of environmental dimensions plus one piece which is related to socio-demographic status:

- Place functions (amenities, density, safety, traffic, objective aesthetics, urban landscape, comfort, and environmental cleanness).
- Place preferences (PP) (crime security, fear of falling, fear of losing, and image).
- Process in environments (cultural environment, social environment, life satisfaction, and sense of belonging).
- Socio-demographic statuses like gender, age, marital status, and occupation.

The objective measures of the environment of neighborhoods were based on the variables of the Geographical Information System (GIS), which were measured in Euclidean or straight-line distances buffer within 500 m of the centroid of a neighborhood using ArcGIS^®^ version 10 (34).

- Street connectivity: Based on the number of real intersections in an area, street connectivity is defined by the number of links between streets divided by the number of street nodes in the buffer area.
- Residential density: The number of dwelling units was divided by the residential land area in this region to obtain this measure.
- Land use mix: Land use mix is the distribution of development among five types of use (i.e., residential, commercial, recreational, industrial, and others).
- Housing quality: This shows whether or not a person’s home is located inside a deteriorated neighborhood.
- Frequency of POS uses: This measure refers to the number of visits older person’s weekly make to POSs (never, once a week, twice a week, more than three times a week).

In the Self-reported Quality of Life (SF-12), the participant’s self-rated health was reported as ‘excellent,’ ‘very good,’ ‘good,’ ‘poor,’ or ‘very poor.’ Physical and mental quality of life was recorded using 12 questions from the simplified Iranian version of Medical Outcomes Study Short Form 12 (SF-12 v2) Health Survey. The survey has already been validated in Iranian populations and is widely used to evaluate the general population’s health (35). According to Montazeri et al. (35), the evaluation of physical health requires a combination of four subscales [general health (one item), physical functioning (two items), physical role (two items), and bodily pain (one item)]. Evaluation of mental health, too, requires a combination of four subscales [vitality (one item), social functioning (one item), emotional role (two items), and mental health (two items)]. These physical and mental health component scores were calculated using normative subscale scores for the Iranian population (35).

Also, we used the “Self-Rated Social Health of Iranians Questionnaire,” which consists of 33 items to evaluate social health (36). The total score was the sum of all 33 items (minimum one and maximum 5 points/item), and the total score lies between 33 and 165, with higher scores indicating better self-reported social health. This scale comprises three domains; community, family, friends, and relatives, with 19, 6, and 8 items for each. Family refers to all household members, and “friends and relatives” refer to those with whom an individual has a close relationship. Other social activities/communications are included under the “community domain.” The psychometric parameters of this scale were verified in the context of the Iranian population (36).

### Data analysis

We applied Partial Least Square Structural Equation Modeling (PLS-SEM) to understand better the influencing factors of the health of older people in POSs’ attributes-regarding social, mental, and physical domains of health-and to develop a conceptual model. PLS-SEM is a causal predictive analysis of both formative and reflective variables (37). It is a common multivariate analysis method for calculating variance-based structural equation models and is widely applied in social sciences (38). Moreover, it also provides an opportunity to resolve the multi-dimensional causal relationships that are otherwise difficult to explore. PLS-SEM can be used to assess the path coefficient (or connection strengths) as parameters of the model estimating effective connectivity. Furthermore, PLS-SEM also handles data distribution using the bootstrapping technique to calculate the significance value for coefficients of the model (pathway).

The proposed model was analyzed using a two-step process. First, the model deals with latent variables (measurement models) that define the association between latent indicators and their manifest variables. Second, an SEM describes the associations between the latent variables. This model explains the relationships between the latent variables and their related manifest variables. A total of 27 factors derived from the literature were recognized as the observed variables and categorized into five groups called exogenous latent constructs. They included the place function factor, place preference factor, process-in-environment factor, the factor related to objective attributes of the outdoor environment, and personal factor.

**Figure 1:**
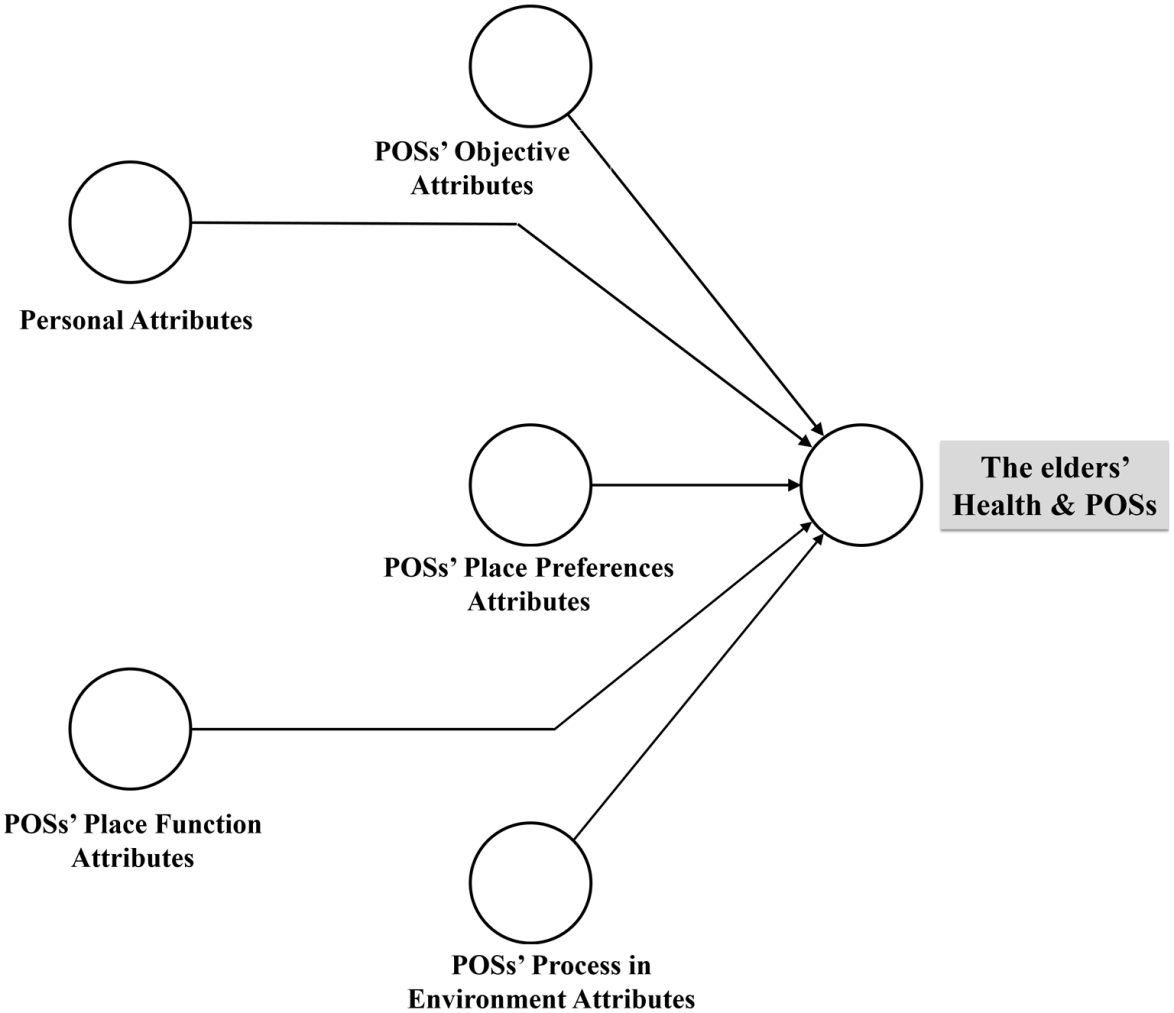
The proposed conceptual framework.

Therefore, the endogenous latent variable (an older person’s health) consisted of five exogenous (observed) variables. Figure 1 shows the conceptual model that illustrates the relationships between endogenous latent constructs and exogenous latent constructs. Therefore, our overarching hypothesis is that five primary constructs influence the health of older adults assessed using physical and mental HRQoL dimensions and social health.

Our research hypotheses are as follows:

*Hypothesis 1 (H1)*. The place function factor (PF) significantly and positively affects older adults’ health.

*Hypothesis 2 (H2)*. The place preference factor (PP) significantly and positively affects older adults’ health.

*Hypothesis 3 (H3)*. The process-in-environment factor (PE) significantly and positively affects older adults’ health.

*Hypothesis 4 (H4)*. The factor related to the outdoor environment objective attributes (OB) significantly and positively affects older adults’ health.

*Hypothesis 5 (H5)*. Personal factor (PR) significantly and positively affects older adults’ health.

To validate the model, the standardized root mean square residual (SRMR) was employed as an index used to show the average of standardized residuals between the observed and the hypothesized covariance matrices (39). SRMR is counted as a measure of the estimated model fit; if SRMR = < 0.08, the study model can be claimed to have a good fit (40), with lower SRMR values indicating a better fit.

## Results

### Baseline data

Data were drawn from the survey conducted on 420 older residents of district 10, Tehran municipality. The study participants’ mean (SD) age was 76.3 ± 9.2 years, 61.3% males, 73.5% married, and 27.2% without a high-school diploma (Table 1). Twenty participants were excluded from the leading models due to high missing data levels.

**Table 1:** Descriptive characteristics of the study participants (*n* = 420).

	Variables	*N* (420)	Percentage
Gender	Men	260	(62%)
	Women	160	(38%)
Marital status	Single	40	(10%)
	Widow	90	(30%)
	Married	290	(60%)
Education	Illiterate / Lower than high school	198	(47%)
	High school	156	(37%)
	Academic	66	(16%)
Occupation	Employed	50	(12%)
	Housewife	122	(29%)
	Retired	248	(59%)
Frequency of being present in POS	Never or once a week	150	(36%)
	Twice a week	150	(36%)
	Three times a week or more	120	(28%)

The simulation to calculate the impact of the observed variables and their latent constructs on the health of older adults was analyzed using smart-PLS version 3.2.7.

### Evaluation of the outer measurement model

The outer measurement model calculates the internal consistency of observed variables (measured by the questionnaire) and their reliability, validity, and unobserved variables (41). Evaluations of consistency are based on a single observed variable and construct reliability tests, while validity is assessed by convergent and discriminant validity (38).

A single observed variable reliability is supposed to describe the variance of an individual observed compared to an unobserved variable by assessing the standardized outer loadings of the observed variables (42). An outer loading of 0.7 or greater means that the observed variables are considered exceedingly acceptable (38), whereas a value of less than 0.7 should be discarded (43). In this study, the cut-off value accepted for the outer loading was 0.7, and 0.4 or greater values are acceptable for exploratory analyses. As listed in Table 2, the outer loadings range between 0.191 and 0.823. Cronbach’s alpha and Composite Reliability (CR) values were used to evaluate internal consistency in construct reliability.

**Table 2:** Construct reliability and validity.

Main constructs	Items	Outer loadings	Cronbach’s alpha	CR	AVE
Place function (PF) factor	PF1: Density	−0.389	0.526	0.722	0.418
	PF2: Amenities	0.633			
	Pf3: Safety	0.556			
	PF4: Urban landscape	0.728			
	PF5: Comfort	0.816			
	PF6: Environmental cleanness	0.670			
	PF7: Aesthetics	0.680			
Place preference (PP) factor	PP1: Security	0.739	0.494	0.704	0.399
	PP2: Fear of falling down	0.859			
	PP3: Way finding	0.383			
	PP4: Perceived aesthetics	0.409			
Process-in-environment (PE) factor	PE1: Social environment	0.621	0.493	0.759	0.347
	PE2: Cultural environment	0.263			
	PE3: Place attachment	0.680			
	PE4: Life satisfaction	0.556			
The factor related to place objective attributes (OB)	OB1: Residential density	0.454	−0.088	0.188	0.235
	OB 2: Land use mix	0.653			
	OB 3: Street connectivity	0.235			
	OB 4: Housing quality	−0.501			
Personal-related factor (PR)	PR1: Age	0.459	0.040	0.223	0.226
	PR2: Education	0.509			
	PR3: Marital status (Married)	0.680			
	PR4: Frequency of visits	0.402			
	PR5: Gender (Female)	−0.191			

Compared with Cronbach’s alpha, CR is supposed to evaluate internal consistency better because it preserves the observed variables’ standardized loadings (44). In our work, however, Cronbach’s alpha and the CR value produced the same results. As seen in Table 2, Cronbach’s alpha was not greater than 0.7, whereas CR for the latent constructs 1, 2, and 3 was more significant than 0.70. Thus, the scales were confirmed as reasonably reliable, indicating that all latent constructs values were more significant than the minimum threshold level of 0.70. The Average Variance Extracted (AVE) of each latent construct was calculated to verify the variables convergent validity (44). The latent constructs in the model should take the lowest 50% of the observed variable variance. This shows that AVE’s value for all the constructs should be greater than 0.5 (45). To achieve discriminant validity, the square root of AVE for each latent variable should be greater than the correlations among the latent variables. So, convergent validity was confirmed for this study model. These results confirmed the convergent validity and the internal consistency of this model.

The next step was to measure the discriminant validity of the latent constructs. The notion of discriminant validity indicates that the manifest variable in any construct should be distinguished from other constructs in the path model in which the cross-loading value in the latent variable is higher than in any other construct (46). We used the Fornell and Larcker criterion and cross-loadings to assess the discriminant validity (44). The proposed standard is that a construct should not indicate the same variance as any other construct more significant than its AVE value (46).

Table 3 lists the Fornell and Larcker criterion test results in which the squared correlations were compared with correlations from other latent constructs. As shown in this table, all correlations were smaller than the squared root of average variance exerted along the diagonals, which implies adequate discriminant validity. All constructs observed variables correspond to the given latent variable, confirming the model’s discriminant validity. As a result of proper reliability, convergent validity, and discriminant validity, the suggested conceptual model proved relatively acceptable.

**Table 3:** Fornell–Larcker criterion test.

Personal-related factor	Objective related factor	Process-in-environment factor	Place preference factor	Place function related factor	
				0.646	Place function related factor
			0.632	0.649	Place preference related factor
		0.589	0.379	0.497	Process-in-environment related factor
	0.484	−0.050	0.005	−0.032	Objective related factor
0.475	−0.197	−0.005	0.013	0.052	Personal-related related factor

### Evaluation of the inner structural model

The validity and reliability of the measurement model were confirmed in this analysis through Inner Structural Model. This entailed an examination of the predictive relevancy of the model and the relationships between the constructs. The primary standards for assessing the inner structural model are the coefficient of determination (*R*^2^), path coefficient (*β* value), and T-statistic.

### Measuring the value of *R*^2^

The coefficient of determination measures the overall effect and variance defined in the endogenous construct for the structural equation model. Therefore, it is a measure of the predictive accuracy of the model. In our study, the inner path model’s value was 0.490 for the quality endogenous latent construct, meaning the five independent constructs can explain 49% of older adults’ health variance. According to Henseler et al. (47) and Hair et al. (46), an *R*^2^ value of 0.75 is conside^r^ed substantial, 0.50 as moderate, and 0.26 as weak. Based on this, the *R*^2^ value in this study was moderate.

### Estimation of path coefficients (β) and T-statistics

The regression analysis showed a similarity between the PLS path coefficients and the standardized β coefficient. The significance of the hypothesis was examined using the *β* value. The β denoted the dependent construct’s expected variation for a unit variation in the independent construct(s). For every path in the hypothesized model, we calculated the *β* values. A more excellent *β* value meant a more substantial effect on the endogenous latent construct. We verified the significant level of *β* value using the T-statistics test. We used the bootstrapping procedure to assess the hypothesis’s significance (48). The importance of the path coefficient and T-statistics values was tested through a bootstrapping method using 5,000 subsamples. Table 4 summarizes our results.

**Table 4:** Path coefficient and T-statistics.

Hypothesized path	Standardized beta	*T*-values	*p*-values	Supported
Place function related factor – > older adults’ health	0.053	0.838	0.402	_
Place preference related factor – > older adults’ health	0.180	3.196	0.01	✓
Process-in-environment related factor – > older adults’ health	0.218	4.715	0.01	✓
Place objective attributes related factor – > older adults’ health	−0.068	0.875	0.382	_
PR related factor – > older adults’ health	0.224	3.196	0.014	✓

As to H1, the PF factor (*β* = 0.053, *T* = 838, *p* > 0.05) did not significantly and positively influence older adults’ health. This was also the case for the OB factor (*β* = −0.068, *T* = 0.875, *p* > 0.05). Therefore, this model could not support H4. As predicted, the findings in Table 4 show that the PR factor significantly influenced health (*β* = 0.224, *T* = 3.194, *p* = 0.014). Thus, H5 was robustly supported. Concerning the direct and positive effect of the PE factor on health (H3), the results in Table 4 confirm that this factor highly significantly affects the health status in older adults (*β* = 0.218, *T* = 4.715, *p* < 0.001), thereby confirming H3. The influence of place preferences (PP factor) on health in older adults was significant and positive (*β* = 0.180, *T* = 3.196, *p* = 0.01), which supports H2.

**Figure 2:**
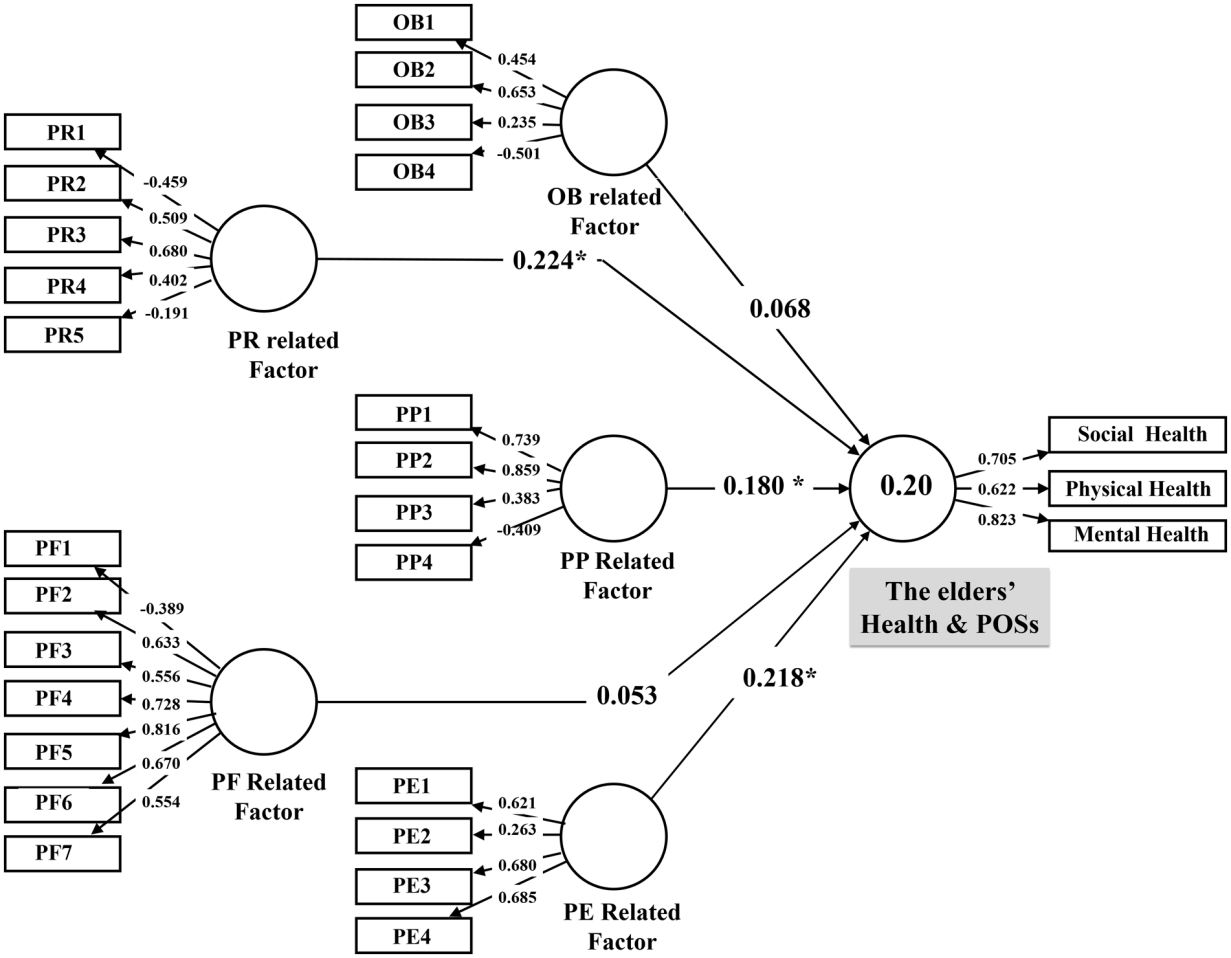
The path model. (Coefficient *p < 0.05).

A more significant beta coefficient (β) among the supported latent variables would indicate a more robust effect from an exogenous latent construct on the endogenous latent construct. Table 4 and Figure 2 show that PF and PE factors had the highest path coefficient (*β* = 0.224 and *β* = 0.218) compared to other *β* values in the model. This indicates that these factors had a more significant variance and a more substantial effect on older people’s health. The PP-related factors had the slightest impact on health (*β* = 0.180).

To Emphasize the model fit, our results indicate that the SRMR of our model was 0.095, showing that the model has moderate goodness to fit. The associated Chi-Square value was 1866.461, and NFI was 0.211.

## Discussion

By adopting an ecological approach, we examined the relationship between the perceived and objective aspects of urban POSs and the health domains of older adults in poor urban neighborhoods of Tehran, Iran. Three health domains (social, mental, and physical) were evaluated based on an individual’s perception of the place, which has its roots in the person-environment relationship, place features (both perceived and objective), and socio-demographic status. Thus, we have hypothesized and proposed a predictive path model. Based on the findings, the physical, mental, and social health in older adults is associated with some attributes of POSs as place preferences (Security, Fear of Falling Down. Way Finding and Perceived Aesthetics) and processes in the environment (Social Environment, Cultural Environment, Place Attachment and Life Satisfaction). Our study also emphasized the role of the personal aspect and socio-demographic status (such as Gender, Marital status, Education, and Occupation) in making older people healthy by using POSs in their neighborhoods, mainly the Frequency of being present in POS.

The predictive model revolves around the relationship between older adults’ health and urban space features. It depends on place function (safety, landscape design, place aesthetics, cleanliness, amenities, and sense of comfort), older adults’ place preferences (security of the environment, subject aesthetics, fear of falling down, and fear of getting lost), and place processes (social background, cultural features, sense of belonging to the place, and life satisfaction). In addition, the model examines the relationship between objective dimensions such as the density of land uses, residential density, the number of intersections in the unit of area, and the quality of the residential environment (new or deteriorated), as well as individual profiles such as gender, age, marital status, and frequency of visits to a POSs and health.

The results suggest that personal features, the process of place-time relationships, and place preferences are related to older adults’ health. Older adults’ health in urban spaces is a function of mental, social, and physical health. In contrast to most studies focusing mainly on physical health (49), the present study shows that social and mental health is more substantial among older adults in deprived areas. Accordingly, mental health’s effect on general health is more vital than physical health (50). Also, for older adults, their physical and psychological health depends on their social health and social relationships.

In agreement with other studies (51), socio-demographic status substantially affects marital status. Education has the closest relationship with health among older adults since they cause individuals to pay more attention to their health and health-related behaviors. Having a spouse decreases the sense of loneliness and abandonment, enhancing one’s health and quality of life. In this study, increasing presence in urban spaces influences older adults’ health. The gender dimension shows that presence in urban spaces only affects the health of males. This is in line with the study on the health of older adults conducted in Japan (12).

Studying the relationship between health and the perceived features of urban space shows that the formation of place function that builds upon non-physical aspects of the environment has a closer relationship with older adults’ health than place preferences and place function. In the non-physical dimension of place, older adults’ most influential health factors are life satisfaction, sense of belonging to the neighborhood and urban space, attachment to the social environment, and cultural factors. The sense of belonging, formed by being rooted in a place and having a place-bound identity, creates stability and mental security in an individual. In addition, older people are usually unwilling to move to another place because they are bound to a place for their memories. Previous studies have mentioned the effect of place attachment on social health (52). Satisfaction with one’s residential environment and belonging to the neighborhood and urban space creates a sense of comfort in older adults. Thus, urban space and local parks in dense neighborhoods act like one’s own courtyard, enhancing a sense of control in older adults (53).

The role of the social environment as an opportunity for social interactions prevents the sense of abandonment and loneliness in older adults. Most studies regarded social relationships as a trigger of trust and support in older adults, increasing mental health and preventing anxiety and distress. Moreover, it will also enhance the social functioning of older adults, thus resulting in better relationships with family members and friends and boosting the sense of belonging in society. Thus, relatedness is the sense of belonging and being a member of the social environment, which was demonstrated to have strong relationships with positive feelings, life purposes, perceived vitality, personal development, and life satisfaction in older people (54).

Cultural factors such as the segregation of men and women and the limited presence of house pets due to Iran’s cultural restrictions have less remarkable effects on older adults’ health (7, 11, 53, 55).

After the process of the person-place relationship, place preferences have the most substantial effect on the relationship between health and urban space. Lack of fear of falling, sense of security, and empirical-affective aesthetics of urban spaces contribute to place preferences on older adults’ health. Fear of falling down is a major preventive factor that depends on the individual’s background (56), which has its roots in older adults’ physical and mental health. The sense of security that results from the low level of crimes and uncivilized behavior can create a sense of comfort in the elderly and make them feel like they are in their courtyard in POSs.

Consistent with our findings, Fuller (57) states that the perceived difficulty of tasks, such as those used to control body posture, has a strong relationship with the prediction of risk, which is indicative of an emotional reflex to dangers, e.g., fear of falling (57). Therefore, anybody with a fear of falling down should limit outdoor physical activities, which, if not considered, eliminates the people with the fear of falling from the group at risk (1). Besides, if this feeling of fear through specially designed environmental spaces decreases, an increase will result in individuals’ physical activity (56, 58), which may promote active aging (59).

Finally, the aesthetic experience of older adults, which results from perceiving harmony and geometry, could result in mental comfort and overall health. According to previous studies, protection against traffic, walking level and safe parks, the intensity of street noise (60), physical barriers, aesthetics, and crime (61) impact the health-related quality of life of older adults. Although previous studies have shown that the objective dimensions of place affect the health of older adults (34, 60, 62), the present study does not confirm such a relationship. In addition, this study does not show any relationship between older adults’ perception of place function and their health.

One limitation of the present study is the restriction of the study scale only to deprived neighborhoods. Furthermore, our study was conducted over 3 months; this can lead to different results if conducted at different time points of the year since it is most likely that older adults’ perception of POSs differs between seasons.

This study’s results can contribute to the design of public environments for older adults to improve their social, mental, and physical health. This makes possible the idea of aging in place through physical and non-physical interventions and providing a high-quality urban environment that meets the older peoples’ sense of belonging and preferences. Our study highlights the importance of appropriate housing and providing POSs tailored to their needs for them to be able to socialize and participate in social-cultural events. Increasing social capital is another way to form social networks and social cohesion. Moreover, our study highlights the need to design spaces without barriers suitable for walking, provide protection against crime, and prepare the environment with fewer falls risk.

## Conclusion

We found positive associations between Place preference, Process-in-environment, and personal health-related factors to elders’ health (social, mental, and physical). Therefore, we recommend that older adults’ preferences concerning outdoor spaces and their life satisfaction and relatedness (i.e., social interaction and place attachment) could be considered for planning and designing outdoor spaces’ features before implementing urban management policies. The path model presented is a suitable approach to using various dimensions of the neighborhood environment, including objective and perceived features.

## Data availability statement

The raw data supporting the conclusions of this article will be made available by the authors, without undue reservation.

## Ethics statement

The studies involving human participants were reviewed and approved by the Iran University of Medical Sciences. The patients/participants provided their written informed consent to participate in this study.

## Author contributions

HB and AL created the study’s concept and design. AL conducted the research, developed the methodology, and authored the article. SK used SPSS to analyze the analysis. PM offered thorough criticism of the manuscript. All authors contributed to the article and approved the submitted version.

## Conflict of interest

The authors declare that the research was conducted in the absence of any commercial or financial relationships that could be construed as a potential conflict of interest.

## Publisher’s note

All claims expressed in this article are solely those of the authors and do not necessarily represent those of their affiliated organizations, or those of the publisher, the editors and the reviewers. Any product that may be evaluated in this article, or claim that may be made by its manufacturer, is not guaranteed or endorsed by the publisher.
